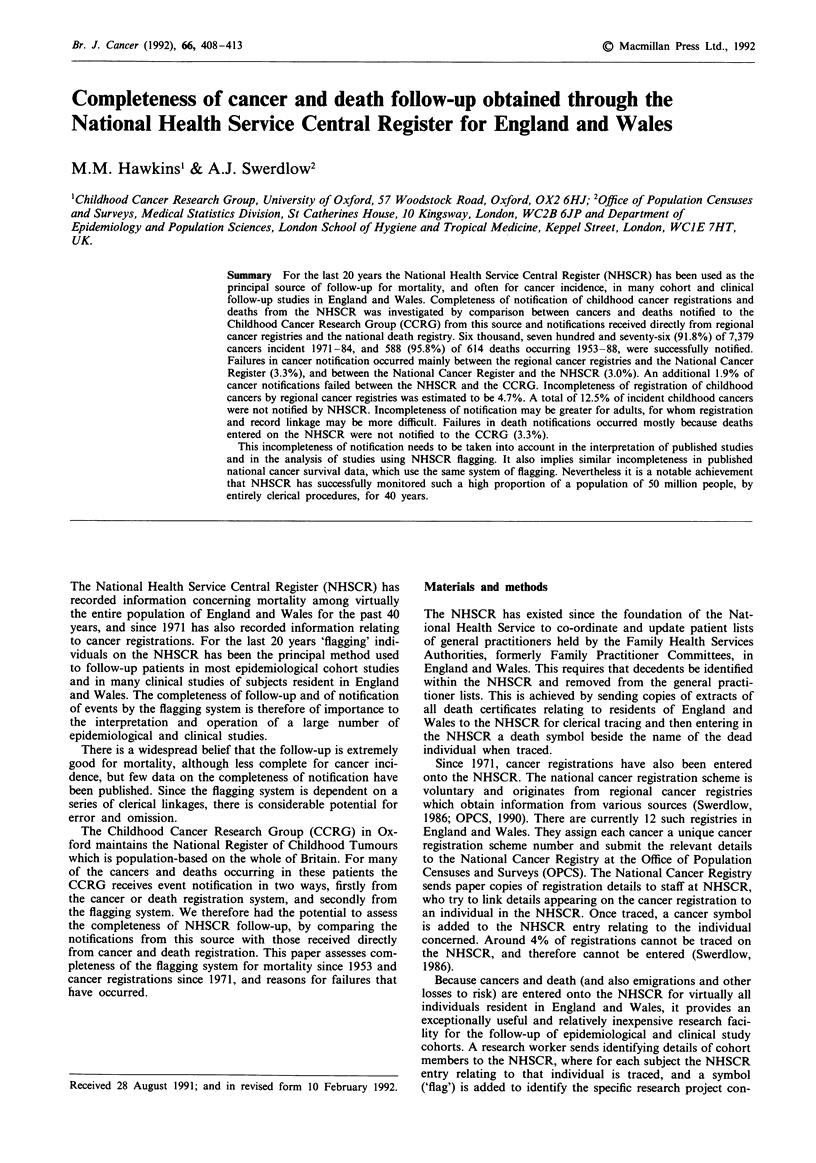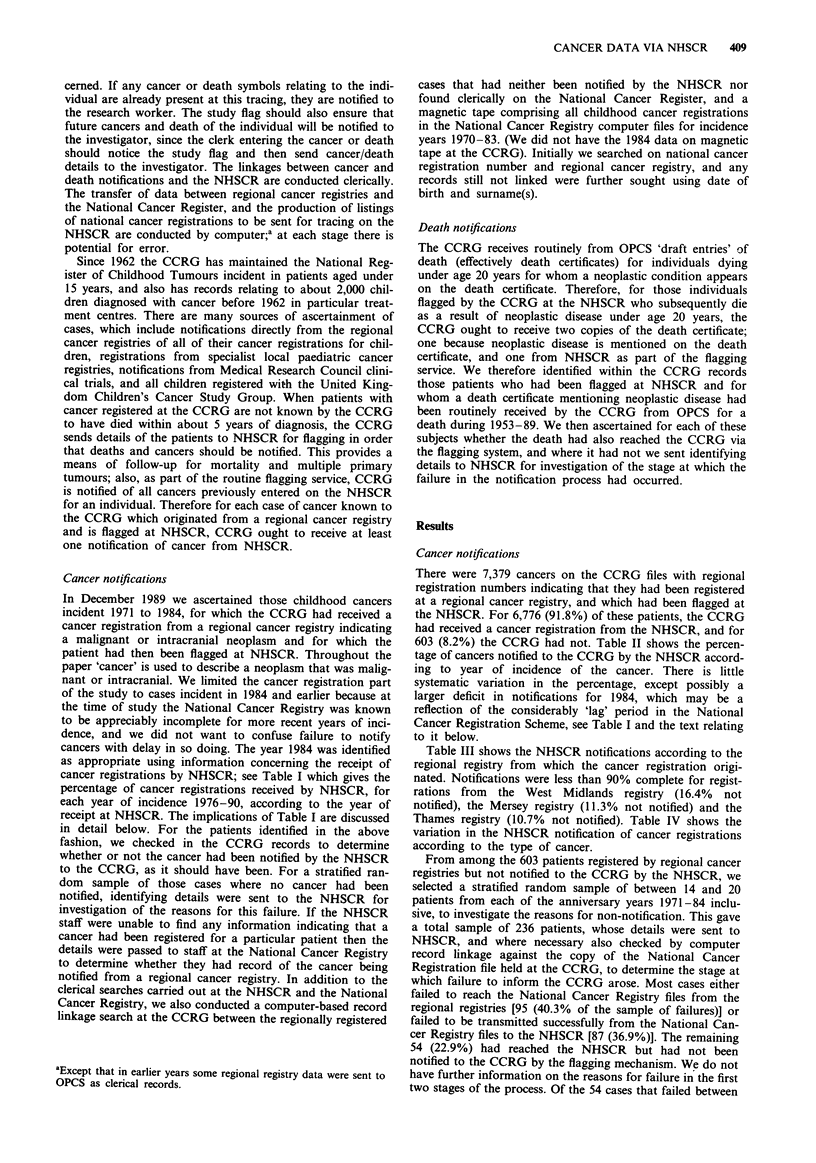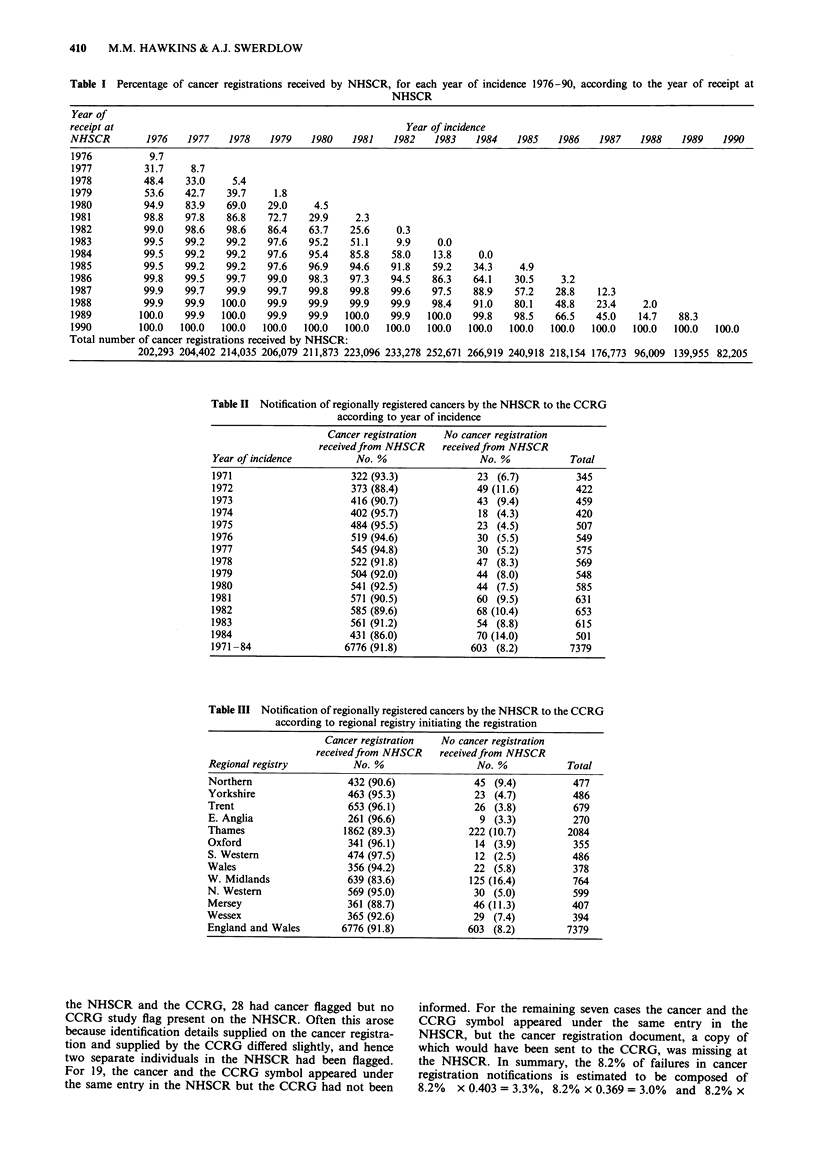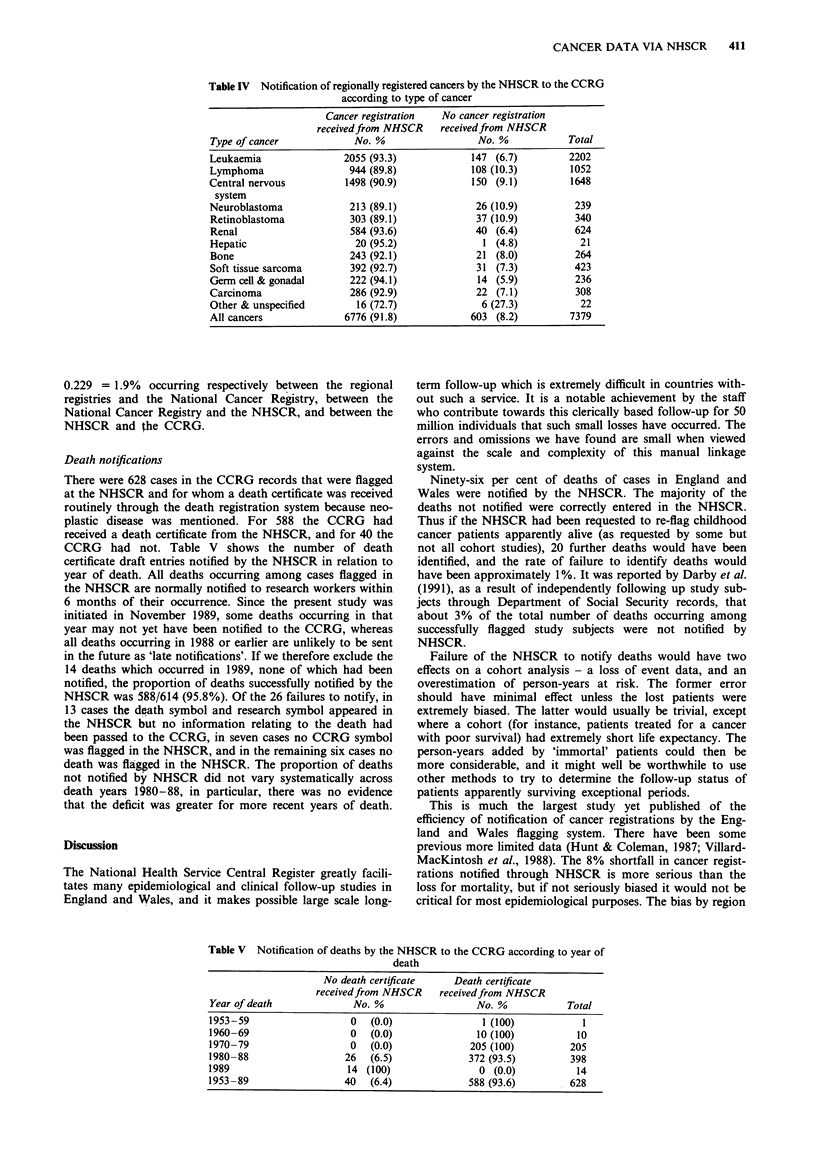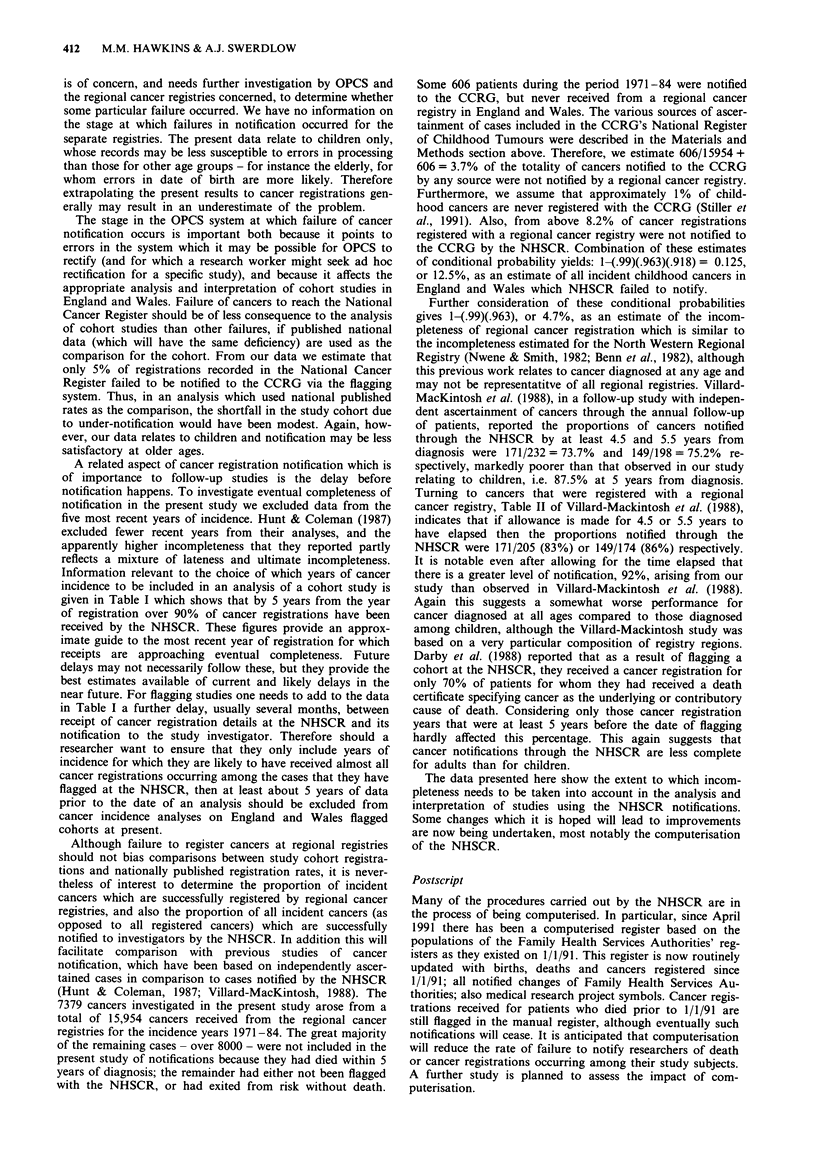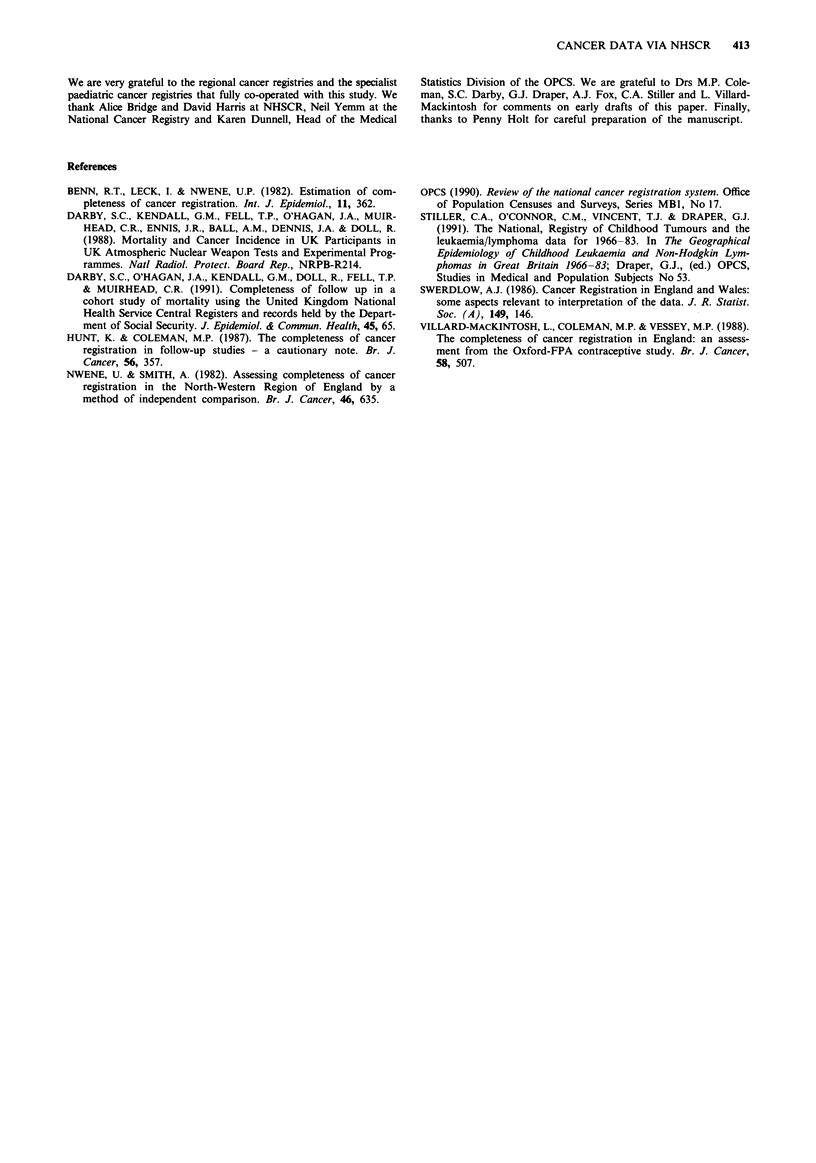# Completeness of cancer and death follow-up obtained through the National Health Service Central Register for England and Wales.

**DOI:** 10.1038/bjc.1992.279

**Published:** 1992-08

**Authors:** M. M. Hawkins, A. J. Swerdlow

**Affiliations:** Childhood Cancer Research Group, University of Oxford, UK.

## Abstract

For the last 20 years the National Health Service Central Register (NHSCR) has been used as the principal source of follow-up for mortality, and often for cancer incidence, in many cohort and clinical follow-up studies in England and Wales. Completeness of notification of childhood cancer registrations and deaths from the NHSCR was investigated by comparison between cancers and deaths notified to the Childhood Cancer Research Group (CCRG) from this source and notifications received directly from regional cancer registries and the national death registry. Six thousand, seven hundred and seventy-six (91.8%) of 7,379 cancers incident 1971-84, and 588 (95.8%) of 614 deaths occurring 1953-88, were successfully notified. Failures in cancer notification occurred mainly between the regional cancer registries and the National Cancer Register (3.3%), and between the National Cancer Register and the NHSCR (3.0%). An additional 1.9% of cancer notifications failed between the NHSCR and the CCRG. Incompleteness of registration of childhood cancers by regional cancer registries was estimated to be 4.7%. A total of 12.5% of incident childhood cancers were not notified by NHSCR. Incompleteness of notification may be greater for adults, for whom registration and record linkage may be more difficult. Failures in death notifications occurred mostly because deaths entered on the NHSCR were not notified to the CCRG (3.3%). This incompleteness of notification needs to be taken into account in the interpretation of published studies and in the analysis of studies using NHSCR flagging. It also implies similar incompleteness in published national cancer survival data, which use the same system of flagging. Nevertheless it is a notable achievement that NHSCR has successfully monitored such a high proportion of a population of 50 million people, by entirely clerical procedures, for 40 years.


Br. J. Cancer (1992), 66, 408-413 © Macmillan Press Ltd., 1992

### Completeness of cancer and death follow-up obtained through the National Health Service Central Register for England and Wales

M.M. Hawkins[1] & A.J. Swerdlow[2]

[1]*Childhood Cancer Research Group, University of Oxford, 57 Woodstock Road, Oxford, OX2 6HJ;* [2]*Office of Population Censuses and Surveys, Medical Statistics Division, St Catherines House, 10 Kingsway, London, WC2B 6JP and Department of Epidemiology and Population Sciences, London School of Hygiene and Tropical Medicine, Keppel Street, London, WC1E 7HT, UK.*

**Summary** For the last 20 years the National Health Service Central Register (NHSCR) has been used as the principal source of follow-up for mortality, and often for cancer incidence, in many cohort and clinical follow-up studies in England and Wales. Completeness of notification of childhood cancer registrations and deaths from the NHSCR was investigated by comparison between cancers and deaths notified to the Childhood Cancer Research Group (CCRG) from this source and notifications received directly from regional cancer registries and the national death registry. Six thousand, seven hundred and seventy-six (91.8%) of 7,379 cancers incident 1971-84, and 588 (95.8%) of 614 deaths occurring 1953-88, were successfully notified. Failures in cancer notification occurred mainly between the regional cancer registries and the National Cancer Register (3.3%), and between the National Cancer Register and the NHSCR (3.0%). An additional 1.9% of cancer notifications failed between the NHSCR and the CCRG. Incompleteness of registration of childhood cancers by regional cancer registries was estimated to be 4.7%. A total of 12.5% of incident childhood cancers were not notified by NHSCR. Incompleteness of notification may be greater for adults, for whom registration and record linkage may be more difficult. Failures in death notifications occurred mostly because deaths entered on the NHSCR were not notified to the CCRG (3.3%).

This incompleteness of notification needs to be taken into account in the interpretation of published studies and in the analysis of studies using NHSCR flagging. It also implies similar incompleteness in published national cancer survival data, which use the same system of flagging. Nevertheless it is a notable achievement that NHSCR has successfully monitored such a high proportion of a population of 50 million people, by entirely clerical procedures, for 40 years.

The National Health Service Central Register (NHSCR) has recorded information concerning mortality among virtually the entire population of England and Wales for the past 40 years, and since 1971 has also recorded information relating to cancer registrations. For the last 20 years 'flagging' individuals on the NHSCR has been the principal method used to follow-up patients in most epidemiological cohort studies and in many clinical studies of subjects resident in England and Wales. The completeness of follow-up and of notification of events by the flagging system is therefore of importance to the interpretation and operation of a large number of epidemiological and clinical studies.

There is a widespread belief that the follow-up is extremely good for mortality, although less complete for cancer incidence, but few data on the completeness of notification have been published. Since the flagging system is dependent on a series of clerical linkages, there is considerable potential for error and omission.

The Childhood Cancer Research Group (CCRG) in Oxford maintains the National Register of Childhood Tumours which is population-based on the whole of Britain. For many of the cancers and deaths occurring in these patients the CCRG receives event notification in two ways, firstly from the cancer or death registration system, and secondly from the flagging system. We therefore had the potential to assess the completeness of NHSCR follow-up, by comparing the notifications from this source with those received directly from cancer and death registration. This paper assesses completeness of the flagging system for mortality since 1953 and cancer registrations since 1971, and reasons for failures that have occurred.

Received 28 August 1991; and in revised form 10 February 1992.

### Materials and methods

The NHSCR has existed since the foundation of the National Health Service to co-ordinate and update patient lists of general practitioners held by the Family Health Services Authorities, formerly Family Practitioner Committees, in England and Wales. This requires that decedents be identified within the NHSCR and removed from the general practitioner lists. This is achieved by sending copies of extracts of all death certificates relating to residents of England and Wales to the NHSCR for clerical tracing and then entering in the NHSCR a death symbol beside the name of the dead individual when traced.

Since 1971, cancer registrations have also been entered onto the NHSCR. The national cancer registration scheme is voluntary and originates from regional cancer registries which obtain information from various sources (Swerdlow, 1986; OPCS, 1990). There are currently 12 such registries in England and Wales. They assign each cancer a unique cancer registration scheme number and submit the relevant details to the National Cancer Registry at the Office of Population Censuses and Surveys (OPCS). The National Cancer Registry sends paper copies of registration details to staff at NHSCR, who try to link details appearing on the cancer registration to an individual in the NHSCR. Once traced, a cancer symbol is added to the NHSCR entry relating to the individual concerned. Around 4% of registrations cannot be traced on the NHSCR, and therefore cannot be entered (Swerdlow, 1986).

Because cancers and death (and also emigrations and other losses to risk) are entered onto the NHSCR for virtually all individuals resident in England and Wales, it provides an exceptionally useful and relatively inexpensive research facility for the follow-up of epidemiological and clinical study cohorts. A research worker sends identifying details of cohort members to the NHSCR, where for each subject the NHSCR entry relating to that individual is traced, and a symbol ('flag') is added to identify the specific research project concerned. If any cancer or death symbols relating to the individual are already present at this tracing, they are notified to the research worker. The study flag should also ensure that future cancers and death of the individual will be notified to the investigator, since the clerk entering the cancer or death should notice the study flag and then send cancer/death details to the investigator. The linkages between cancer and death notifications and the NHSCR are conducted clerically. The transfer of data between regional cancer registries and the National Cancer Register, and the production of listings of national cancer registrations to be sent for tracing on the NHSCR are conducted by computer;[a] at each stage there is potential for error.

Since 1962 the CCRG has maintained the National Register of Childhood Tumours incident in patients aged under 15 years, and also has records relating to about 2,000 children diagnosed with cancer before 1962 in particular treatment centres. There are many sources of ascertainment of cases, which include notifications directly from the regional cancer registries of all of their cancer registrations for children, registrations from specialist local paediatric cancer registries, notifications from Medical Research Council clinical trials, and all children registered with the United Kingdom Children's Cancer Study Group. When patients with cancer registered at the CCRG are not known by the CCRG to have died within about 5 years of diagnosis, the CCRG sends details of the patients to NHSCR for flagging in order that deaths and cancers should be notified. This provides a means of follow-up for mortality and multiple primary tumours; also, as part of the routine flagging service, CCRG is notified of all cancers previously entered on the NHSCR for an individual. Therefore for each case of cancer known to the CCRG which originated from a regional cancer registry and is flagged at NHSCR, CCRG ought to receive at least one notification of cancer from NHSCR.

*Cancer notifications*

In December 1989 we ascertained those childhood cancers incident 1971 to 1984, for which the CCRG had received a cancer registration from a regional cancer registry indicating a malignant or intracranial neoplasm and for which the patient had then been flagged at NHSCR. Throughout the paper 'cancer' is used to describe a neoplasm that was malignant or intracranial. We limited the cancer registration part of the study to cases incident in 1984 and earlier because at the time of study the National Cancer Registry was known to be appreciably incomplete for more recent years of incidence, and we did not want to confuse failure to notify cancers with delay in so doing. The year 1984 was identified as appropriate using information concerning the receipt of cancer registrations by NHSCR; see Table I which gives the percentage of cancer registrations received by NHSCR, for each year of incidence 1976–90, according to the year of receipt at NHSCR. The implications of Table I are discussed in detail below. For the patients identified in the above fashion, we checked in the CCRG records to determine whether or not the cancer had been notified by the NHSCR to the CCRG, as it should have been. For a stratified random sample of those cases where no cancer had been notified, identifying details were sent to the NHSCR for investigation of the reasons for this failure. If the NHSCR staff were unable to find any information indicating that a cancer had been registered for a particular patient then the details were passed to staff at the National Cancer Registry to determine whether they had record of the cancer being notified from a regional cancer registry. In addition to the clerical searches carried out at the NHSCR and the National Cancer Registry, we also conducted a computer-based record linkage search at the CCRG between the regionally registered cases that had neither been notified by the NHSCR nor found clerically on the National Cancer Register, and a magnetic tape comprising all childhood cancer registrations in the National Cancer Registry computer files for incidence years 1970–83. (We did not have the 1984 data on magnetic tape at the CCRG). Initially we searched on national cancer registration number and regional cancer registry, and any records still not linked were further sought using date of birth and surname(s).

[a]Except that in earlier years some regional registry data were sent to OPCS as clerical records.

*Death notifications*

The CCRG receives routinely from OPCS 'draft entries' of death (effectively death certificates) for individuals dying under age 20 years for whom a neoplastic condition appears on the death certificate. Therefore, for those individuals flagged by the CCRG at the NHSCR who subsequently die as a result of neoplastic disease under age 20 years, the CCRG ought to receive two copies of the death certificate; one because neoplastic disease is mentioned on the death certificate, and one from NHSCR as part of the flagging service. We therefore identified within the CCRG records those patients who had been flagged at NHSCR and for whom a death certificate mentioning neoplastic disease had been routinely received by the CCRG from OPCS for a death during 1953–89. We then ascertained for each of these subjects whether the death had also reached the CCRG via the flagging system, and where it had not we sent identifying details to NHSCR for investigation of the stage at which the failure in the notification process had occurred.

### Results

*Cancer notifications*

There were 7,379 cancers on the CCRG files with regional registration numbers indicating that they had been registered at a regional cancer registry, and which had been flagged at the NHSCR. For 6,776 (91.8%) of these patients, the CCRG had received a cancer registration from the NHSCR, and for 603 (8.2%) the CCRG had not. Table II shows the percentage of cancers notified to the CCRG by the NHSCR according to year of incidence of the cancer. There is little systematic variation in the percentage, except possibly a larger deficit in notifications for 1984, which may be a reflection of the considerably 'lag' period in the National Cancer Registration Scheme, see Table I and the text relating to it below.

Table III shows the NHSCR notifications according to the regional registry from which the cancer registration originated. Notifications were less than 90% complete for registrations from the West Midlands registry (16.4% not notified), the Mersey registry (11.3% not notified) and the Thames registry (10.7% not notified). Table IV shows the variation in the NHSCR notification of cancer registrations according to the type of cancer.

From among the 603 patients registered by regional cancer registries but not notified to the CCRG by the NHSCR, we selected a stratified random sample of between 14 and 20 patients from each of the anniversary years 1971–84 inclusive, to investigate the reasons for non-notification. This gave a total sample of 236 patients, whose details were sent to NHSCR, and where necessary also checked by computer record linkage against the copy of the National Cancer Registration file held at the CCRG, to determine the stage at which failure to inform the CCRG arose. Most cases either failed to reach the National Cancer Registry files from the regional registries [95 (40.3% of the sample of failures)] or failed to be transmitted successfully from the National Cancer Registry files to the NHSCR [87 (36.9%)]. The remaining 54 (22.9%) had reached the NHSCR but had not been notified to the CCRG by the flagging mechanism. We do not have further information on the reasons for failure in the first two stages of the process. Of the 54 cases that failed between

**Table I** Percentage of cancer registrations received by NHSCR, for each year of incidence 1976–90, according to the year of receipt at NHSCR

| *Year of receipt at NHSCR* | *Year of incidence* 1976 | 1977 | 1978 | 1979 | 1980 | 1981 | 1982 | 1983 | 1984 | 1985 | 1986 | 1987 | 1988 | 1989 | 1990 |
|---|---|---|---|---|---|---|---|---|---|---|---|---|---|---|---|
| 1976 | 9.7 | | | | | | | | | | | | | | |
| 1977 | 31.7 | 8.7 | | | | | | | | | | | | | |
| 1978 | 48.4 | 33.0 | 5.4 | | | | | | | | | | | | |
| 1979 | 53.6 | 42.7 | 39.7 | 1.8 | | | | | | | | | | | |
| 1980 | 94.9 | 83.9 | 69.0 | 29.0 | 4.5 | | | | | | | | | | |
| 1981 | 98.8 | 97.8 | 86.8 | 72.7 | 29.9 | 2.3 | | | | | | | | | |
| 1982 | 99.0 | 98.6 | 98.6 | 86.4 | 63.7 | 25.6 | 0.3 | | | | | | | | |
| 1983 | 99.5 | 99.2 | 99.2 | 97.6 | 95.2 | 51.1 | 9.9 | 0.0 | | | | | | | |
| 1984 | 99.5 | 99.2 | 99.2 | 97.6 | 95.4 | 85.8 | 58.0 | 13.8 | 0.0 | | | | | | |
| 1985 | 99.5 | 99.2 | 99.2 | 97.6 | 96.9 | 94.6 | 91.8 | 59.2 | 34.3 | 4.9 | | | | | |
| 1986 | 99.8 | 99.5 | 99.7 | 99.0 | 98.3 | 97.3 | 94.5 | 86.3 | 64.1 | 30.5 | 3.2 | | | | |
| 1987 | 99.9 | 99.7 | 99.9 | 99.7 | 99.8 | 99.8 | 99.6 | 97.5 | 88.9 | 57.2 | 28.8 | 12.3 | | | |
| 1988 | 99.9 | 99.9 | 100.0 | 99.9 | 99.9 | 99.9 | 99.9 | 98.4 | 91.0 | 80.1 | 48.8 | 23.4 | 2.0 | | |
| 1989 | 100.0 | 99.9 | 100.0 | 99.9 | 99.9 | 100.0 | 99.9 | 100.0 | 99.8 | 98.5 | 66.5 | 45.0 | 14.7 | 88.3 | |
| 1990 | 100.0 | 100.0 | 100.0 | 100.0 | 100.0 | 100.0 | 100.0 | 100.0 | 100.0 | 100.0 | 100.0 | 100.0 | 100.0 | 100.0 | 100.0 |
| Total number of cancer registrations received by NHSCR: | 202,293 | 204,402 | 214,035 | 206,079 | 211,873 | 223,096 | 233,278 | 252,671 | 266,919 | 240,918 | 218,154 | 176,773 | 96,009 | 139,955 | 82,205 |

**Table II** Notification of regionally registered cancers by the NHSCR to the CCRG according to year of incidence

| *Year of incidence* | *Cancer registration received from NHSCR No. %* | *No cancer registration received from NHSCR No. %* | *Total* |
|---|---|---|---|
| 1971 | 322 (93.3) | 23 (6.7) | 345 |
| 1972 | 373 (88.4) | 49 (11.6) | 422 |
| 1973 | 416 (90.7) | 43 (9.4) | 459 |
| 1974 | 402 (95.7) | 18 (4.3) | 420 |
| 1975 | 484 (95.5) | 23 (4.5) | 507 |
| 1976 | 519 (94.6) | 30 (5.5) | 549 |
| 1977 | 545 (94.8) | 30 (5.2) | 575 |
| 1978 | 522 (91.8) | 47 (8.3) | 569 |
| 1979 | 504 (92.0) | 44 (8.0) | 548 |
| 1980 | 541 (92.5) | 44 (7.5) | 585 |
| 1981 | 571 (90.5) | 60 (9.5) | 631 |
| 1982 | 585 (89.6) | 68 (10.4) | 653 |
| 1983 | 561 (91.2) | 54 (8.8) | 615 |
| 1984 | 431 (86.0) | 70 (14.0) | 501 |
| 1971–84 | 6776 (91.8) | 603 (8.2) | 7379 |

**Table III** Notification of regionally registered cancers by the NHSCR to the CCRG according to regional registry initiating the registration

| *Regional registry* | *Cancer registration received from NHSCR No. %* | *No cancer registration received from NHSCR No. %* | *Total* |
|---|---|---|---|
| Northern | 432 (90.6) | 45 (9.4) | 477 |
| Yorkshire | 463 (95.3) | 23 (4.7) | 486 |
| Trent | 653 (96.1) | 26 (3.8) | 679 |
| E. Anglia | 261 (96.6) | 9 (3.3) | 270 |
| Thames | 1862 (89.3) | 222 (10.7) | 2084 |
| Oxford | 341 (96.1) | 14 (3.9) | 355 |
| S. Western | 474 (97.5) | 12 (2.5) | 486 |
| Wales | 356 (94.2) | 22 (5.8) | 378 |
| W. Midlands | 639 (83.6) | 125 (16.4) | 764 |
| N. Western | 569 (95.0) | 30 (5.0) | 599 |
| Mersey | 361 (88.7) | 46 (11.3) | 407 |
| Wessex | 365 (92.6) | 29 (7.4) | 394 |
| England and Wales | 6776 (91.8) | 603 (8.2) | 7379 |

the NHSCR and the CCRG, 28 had cancer flagged but no CCRG study flag present on the NHSCR. Often this arose because identification details supplied on the cancer registration and supplied by the CCRG differed slightly, and hence two separate individuals in the NHSCR had been flagged. For 19, the cancer and the CCRG symbol appeared under the same entry in the NHSCR but the CCRG had not been informed. For the remaining seven cases the cancer and the CCRG symbol appeared under the same entry in the NHSCR, but the cancer registration document, a copy of which would have been sent to the CCRG, was missing at the NHSCR. In summary, the 8.2% of failures in cancer registration notifications is estimated to be composed of 8.2% $\times$ 0.403 = 3.3%, 8.2% $\times$ 0.369 = 3.0% and 8.2% $\times$

**Table IV** Notification of regionally registered cancers by the NHSCR to the CCRG according to type of cancer

| *Type of cancer* | *Cancer registration received from NHSCR* No. % | *No cancer registration received from NHSCR* No. % | *Total* |
|---|---|---|---|
| Leukaemia | 2055 (93.3) | 147 (6.7) | 2202 |
| Lymphoma | 944 (89.8) | 108 (10.3) | 1052 |
| Central nervous system | 1498 (90.9) | 150 (9.1) | 1648 |
| Neuroblastoma | 213 (89.1) | 26 (10.9) | 239 |
| Retinoblastoma | 303 (89.1) | 37 (10.9) | 340 |
| Renal | 584 (93.6) | 40 (6.4) | 624 |
| Hepatic | 20 (95.2) | 1 (4.8) | 21 |
| Bone | 243 (92.1) | 21 (8.0) | 264 |
| Soft tissue sarcoma | 392 (92.7) | 31 (7.3) | 423 |
| Germ cell & gonadal | 222 (94.1) | 14 (5.9) | 236 |
| Carcinoma | 286 (92.9) | 22 (7.1) | 308 |
| Other & unspecified | 16 (72.7) | 6 (27.3) | 22 |
| All cancers | 6776 (91.8) | 603 (8.2) | 7379 |

0.229 = 1.9% occurring respectively between the regional registries and the National Cancer Registry, between the National Cancer Registry and the NHSCR, and between the NHSCR and the CCRG.

*Death notifications*

There were 628 cases in the CCRG records that were flagged at the NHSCR and for whom a death certificate was received routinely through the death registration system because neoplastic disease was mentioned. For 588 the CCRG had received a death certificate from the NHSCR, and for 40 the CCRG had not. Table V shows the number of death certificate draft entries notified by the NHSCR in relation to year of death. All deaths occurring among cases flagged in the NHSCR are normally notified to research workers within 6 months of their occurrence. Since the present study was initiated in November 1989, some deaths occurring in that year may not yet have been notified to the CCRG, whereas all deaths occurring in 1988 or earlier are unlikely to be sent in the future as 'late notifications'. If we therefore exclude the 14 deaths which occurred in 1989, none of which had been notified, the proportion of deaths successfully notified by the NHSCR was 588/614 (95.8%). Of the 26 failures to notify, in 13 cases the death symbol and research symbol appeared in the NHSCR but no information relating to the death had been passed to the CCRG, in seven cases no CCRG symbol was flagged in the NHSCR, and in the remaining six cases no death was flagged in the NHSCR. The proportion of deaths not notified by NHSCR did not vary systematically across death years 1980–88, in particular, there was no evidence that the deficit was greater for more recent years of death.

### Discussion

The National Health Service Central Register greatly facilitates many epidemiological and clinical follow-up studies in England and Wales, and it makes possible large scale long-term follow-up which is extremely difficult in countries without such a service. It is a notable achievement by the staff who contribute towards this clerically based follow-up for 50 million individuals that such small losses have occurred. The errors and omissions we have found are small when viewed against the scale and complexity of this manual linkage system.

Ninety-six per cent of deaths of cases in England and Wales were notified by the NHSCR. The majority of the deaths not notified were correctly entered in the NHSCR. Thus if the NHSCR had been requested to re-flag childhood cancer patients apparently alive (as requested by some but not all cohort studies), 20 further deaths would have been identified, and the rate of failure to identify deaths would have been approximately 1%. It was reported by Darby *et al.* (1991), as a result of independently following up study subjects through Department of Social Security records, that about 3% of the total number of deaths occurring among successfully flagged study subjects were not notified by NHSCR.

Failure of the NHSCR to notify deaths would have two effects on a cohort analysis – a loss of event data, and an overestimation of person-years at risk. The former error should have minimal effect unless the lost patients were extremely biased. The latter would usually be trivial, except where a cohort (for instance, patients treated for a cancer with poor survival) had extremely short life expectancy. The person-years added by 'immortal' patients could then be more considerable, and it might well be worthwhile to use other methods to try to determine the follow-up status of patients apparently surviving exceptional periods.

This is much the largest study yet published of the efficiency of notification of cancer registrations by the England and Wales flagging system. There have been some previous more limited data (Hunt & Coleman, 1987; Villard-MacKintosh *et al.*, 1988). The 8% shortfall in cancer registrations notified through NHSCR is more serious than the loss for mortality, but if not seriously biased it would not be critical for most epidemiological purposes. The bias by region

**Table V** Notification of deaths by the NHSCR to the CCRG according to year of death

| *Year of death* | *No death certificate received from NHSCR* No. % | *Death certificate received from NHSCR* No. % | *Total* |
|---|---|---|---|
| 1953–59 | 0 (0.0) | 1 (100) | 1 |
| 1960–69 | 0 (0.0) | 10 (100) | 10 |
| 1970–79 | 0 (0.0) | 205 (100) | 205 |
| 1980–88 | 26 (6.5) | 372 (93.5) | 398 |
| 1989 | 14 (100) | 0 (0.0) | 14 |
| 1953–89 | 40 (6.4) | 588 (93.6) | 628 |

is of concern, and needs further investigation by OPCS and the regional cancer registries concerned, to determine whether some particular failure occurred. We have no information on the stage at which failures in notification occurred for the separate registries. The present data relate to children only, whose records may be less susceptible to errors in processing than those for other age groups – for instance the elderly, for whom errors in date of birth are more likely. Therefore extrapolating the present results to cancer registrations generally may result in an underestimate of the problem.

The stage in the OPCS system at which failure of cancer notification occurs is important both because it points to errors in the system which it may be possible for OPCS to rectify (and for which a research worker might seek ad hoc rectification for a specific study), and because it affects the appropriate analysis and interpretation of cohort studies in England and Wales. Failure of cancers to reach the National Cancer Register should be of less consequence to the analysis of cohort studies than other failures, if published national data (which will have the same deficiency) are used as the comparison for the cohort. From our data we estimate that only 5% of registrations recorded in the National Cancer Register failed to be notified to the CCRG via the flagging system. Thus, in an analysis which used national published rates as the comparison, the shortfall in the study cohort due to under-notification would have been modest. Again, however, our data relates to children and notification may be less satisfactory at older ages.

A related aspect of cancer registration notification which is of importance to follow-up studies is the delay before notification happens. To investigate eventual completeness of notification in the present study we excluded data from the five most recent years of incidence. Hunt & Coleman (1987) excluded fewer recent years from their analyses, and the apparently higher incompleteness that they reported partly reflects a mixture of lateness and ultimate incompleteness. Information relevant to the choice of which years of cancer incidence to be included in an analysis of a cohort study is given in Table I which shows that by 5 years from the year of registration over 90% of cancer registrations have been received by the NHSCR. These figures provide an approximate guide to the most recent year of registration for which receipts are approaching eventual completeness. Future delays may not necessarily follow these, but they provide the best estimates available of current and likely delays in the near future. For flagging studies one needs to add to the data in Table I a further delay, usually several months, between receipt of cancer registration details at the NHSCR and its notification to the study investigator. Therefore should a researcher want to ensure that they only include years of incidence for which they are likely to have received almost all cancer registrations occurring among the cases that they have flagged at the NHSCR, then at least about 5 years of data prior to the date of an analysis should be excluded from cancer incidence analyses on England and Wales flagged cohorts at present.

Although failure to register cancers at regional registries should not bias comparisons between study cohort registrations and nationally published registration rates, it is nevertheless of interest to determine the proportion of incident cancers which are successfully registered by regional cancer registries, and also the proportion of all incident cancers (as opposed to all registered cancers) which are successfully notified to investigators by the NHSCR. In addition this will facilitate comparison with previous studies of cancer notification, which have been based on independently ascertained cases in comparison to cases notified by the NHSCR (Hunt & Coleman, 1987; Villard-MacKintosh, 1988). The 7379 cancers investigated in the present study arose from a total of 15,954 cancers received from the regional cancer registries for the incidence years 1971–84. The great majority of the remaining cases – over 8000 – were not included in the present study of notifications because they had died within 5 years of diagnosis; the remainder had either not been flagged with the NHSCR, or had exited from risk without death. Some 606 patients during the period 1971–84 were notified to the CCRG, but never received from a regional cancer registry in England and Wales. The various sources of ascertainment of cases included in the CCRG's National Register of Childhood Tumours were described in the Materials and Methods section above. Therefore, we estimate 606/15954 + 606 = 3.7% of the totality of cancers notified to the CCRG by any source were not notified by a regional cancer registry. Furthermore, we assume that approximately 1% of childhood cancers are never registered with the CCRG (Stiller *et al.*, 1991). Also, from above 8.2% of cancer registrations registered with a regional cancer registry were not notified to the CCRG by the NHSCR. Combination of these estimates of conditional probability yields: 1–(.99)(.963)(.918) = 0.125, or 12.5%, as an estimate of all incident childhood cancers in England and Wales which NHSCR failed to notify.

Further consideration of these conditional probabilities gives 1–(.99)(.963), or 4.7%, as an estimate of the incompleteness of regional cancer registration which is similar to the incompleteness estimated for the North Western Regional Registry (Nwene & Smith, 1982; Benn *et al.*, 1982), although this previous work relates to cancer diagnosed at any age and may not be representatitve of all regional registries. Villard-MacKintosh *et al.* (1988), in a follow-up study with independent ascertainment of cancers through the annual follow-up of patients, reported the proportions of cancers notified through the NHSCR by at least 4.5 and 5.5 years from diagnosis were 171/232 = 73.7% and 149/198 = 75.2% respectively, markedly poorer than that observed in our study relating to children, i.e. 87.5% at 5 years from diagnosis. Turning to cancers that were registered with a regional cancer registry, Table II of Villard-Mackintosh *et al.* (1988), indicates that if allowance is made for 4.5 or 5.5 years to have elapsed then the proportions notified through the NHSCR were 171/205 (83%) or 149/174 (86%) respectively. It is notable even after allowing for the time elapsed that there is a greater level of notification, 92%, arising from our study than observed in Villard-Mackintosh *et al.* (1988). Again this suggests a somewhat worse performance for cancer diagnosed at all ages compared to those diagnosed among children, although the Villard-Mackintosh study was based on a very particular composition of registry regions. Darby *et al.* (1988) reported that as a result of flagging a cohort at the NHSCR, they received a cancer registration for only 70% of patients for whom they had received a death certificate specifying cancer as the underlying or contributory cause of death. Considering only those cancer registration years that were at least 5 years before the date of flagging hardly affected this percentage. This again suggests that cancer notifications through the NHSCR are less complete for adults than for children.

The data presented here show the extent to which incompleteness needs to be taken into account in the analysis and interpretation of studies using the NHSCR notifications. Some changes which it is hoped will lead to improvements are now being undertaken, most notably the computerisation of the NHSCR.

### *Postscript*

Many of the procedures carried out by the NHSCR are in the process of being computerised. In particular, since April 1991 there has been a computerised register based on the populations of the Family Health Services Authorities' registers as they existed on 1/1/91. This register is now routinely updated with births, deaths and cancers registered since 1/1/91; all notified changes of Family Health Services Authorities; also medical research project symbols. Cancer registrations received for patients who died prior to 1/1/91 are still flagged in the manual register, although eventually such notifications will cease. It is anticipated that computerisation will reduce the rate of failure to notify researchers of death or cancer registrations occurring among their study subjects. A further study is planned to assess the impact of computerisation.

We are very grateful to the regional cancer registries and the specialist paediatric cancer registries that fully co-operated with this study. We thank Alice Bridge and David Harris at NHSCR, Neil Yemm at the National Cancer Registry and Karen Dunnell, Head of the Medical Statistics Division of the OPCS. We are grateful to Drs M.P. Coleman, S.C. Darby, G.J. Draper, A.J. Fox, C.A. Stiller and L. Villard-Mackintosh for comments on early drafts of this paper. Finally, thanks to Penny Holt for careful preparation of the manuscript.

### References

BENN, R.T., LECK, I. & NWENE, U.P. (1982). Estimation of completeness of cancer registration. *Int. J. Epidemiol.*, **11**, 362.

DARBY, S.C., KENDALL, G.M., FELL, T.P., O'HAGAN, J.A., MUIRHEAD, C.R., ENNIS, J.R., BALL, A.M., DENNIS, J.A. & DOLL, R. (1988). Mortality and Cancer Incidence in UK Participants in UK Atmospheric Nuclear Weapon Tests and Experimental Programmes. *Natl Radiol. Protect. Board Rep.*, NRPB-R214.

DARBY, S.C., O'HAGAN, J.A., KENDALL, G.M., DOLL, R., FELL, T.P. & MUIRHEAD, C.R. (1991). Completeness of follow up in a cohort study of mortality using the United Kingdom National Health Service Central Registers and records held by the Department of Social Security. *J. Epidemiol. & Commun. Health*, **45**, 65.

HUNT, K. & COLEMAN, M.P. (1987). The completeness of cancer registration in follow-up studies – a cautionary note. *Br. J. Cancer*, **56**, 357.

NWENE, U. & SMITH, A. (1982). Assessing completeness of cancer registration in the North-Western Region of England by a method of independent comparison. *Br. J. Cancer*, **46**, 635.

OPCS (1990). *Review of the national cancer registration system.* Office of Population Censuses and Surveys, Series MB1, No 17.

STILLER, C.A., O'CONNOR, C.M., VINCENT, T.J. & DRAPER, G.J. (1991). The National, Registry of Childhood Tumours and the leukaemia/lymphoma data for 1966–83. In *The Geographical Epidemiology of Childhood Leukaemia and Non-Hodgkin Lymphomas in Great Britain 1966–83*; Draper, G.J., (ed.) OPCS, Studies in Medical and Population Subjects No 53.

SWERDLOW, A.J. (1986). Cancer Registration in England and Wales: some aspects relevant to interpretation of the data. *J. R. Statist. Soc. (A)*, **149**, 146.

VILLARD-MACKINTOSH, L., COLEMAN, M.P. & VESSEY, M.P. (1988). The completeness of cancer registration in England: an assessment from the Oxford-FPA contraceptive study. *Br. J. Cancer*, **58**, 507.